# Prognostic performance of baseline serum immunoglobulin G in patients with metastatic renal cell carcinoma receiving first-line ICI–TKI therapy

**DOI:** 10.3389/fonc.2026.1928984

**Published:** 2026-09-21

**Authors:** Hongwei Wang, Xingang Bi, Tian Han, Gan Du, Youyan Guan, Aiping Zhou, Changling Li, Jianzhong Shou, Hongzhe Shi

**Affiliations:** 1 Department of Urology, National Cancer Center/National Clinical Research Center for Cancer/Cancer Hospital, Chinese Academy of Medical Sciences and Peking Union Medical College, Beijing, China; 2 Department of Medical Oncology, National Cancer Center/National Clinical Research Center for Cancer/Cancer Hospital, Chinese Academy of Medical Sciences and Peking Union Medical College, Beijing, China

**Keywords:** immunoglobulin G, immunotherapy, metastatic renal cell carcinoma, prognostic biomarker, targeted therapy

## Abstract

**Background:**

In the era of immune checkpoint inhibitor–tyrosine kinase inhibitor (ICI–TKI) combination therapy for metastatic renal cell carcinoma (mRCC), reliable biomarkers for prognostic stratification and long-term prognosis remain clinically unmet needs. This study aimed to investigate the prognostic significance of baseline serum IgG in patients with mRCC receiving first-line systemic therapy and to evaluate its potential in complementing current risk stratification models.

**Methods:**

We retrospectively analyzed 153 patients with mRCC receiving first-line ICI-TKI therapy between May 2019 and April 2026. Baseline serum IgG levels were categorized as elevated (>17.4 g/L) or normal based on the institutional reference limit. Survival outcomes were assessed using Kaplan–Meier analysis and Cox proportional hazards models. A prognostic nomogram was developed for individualized survival prediction.

**Results:**

The median follow-up was 29.0 months. Elevated baseline IgG was associated with higher IMDC risk (P = 0.022). Kaplan–Meier analysis showed significantly worse overall survival (OS; P < 0.001) and progression-free survival (PFS; P = 0.049) in patients with elevated IgG. In multivariable analysis, elevated IgG was not associated with PFS but remained an independent predictor of inferior OS (HR = 2.55; 95% CI, 1.30–5.00; P = 0.006). Incorporating IgG into the IMDC model improved the C-index from 0.645 to 0.689, with the greatest incremental benefit observed for 3-year OS prediction. The exploratory nomogram demonstrated acceptable calibration, particularly for 3-year OS prediction.

**Conclusions:**

Baseline serum IgG is an independent prognostic biomarker for OS in mRCC patients treated with first-line ICI–TKI therapy. Its integration into the IMDC model improves survival prediction and provides additional prognostic information in the immunotherapy era.

## 1 Background

Renal cell carcinoma (RCC) represents a significant global health burden, currently ranked as the 14th most common malignancy worldwide. Despite substantial advances in systemic therapies, mRCC remains associated with considerable morbidity and mortality, posing a major clinical challenge worldwide (1–3). Over the past decade, the management landscape for mRCC has undergone an unprecedented transformation, transitioning from cytokine-based therapies and VEGF-targeted tyrosine kinase inhibitors (TKIs) to immune checkpoint inhibitor (ICI)-based combinations (4, 5). These advancements have shifted clinical focus toward identifying robust biomarkers capable of refining prognostic stratification and informing clinical decision-making. While the International Metastatic Renal Cell Carcinoma Database Consortium (IMDC) risk model remains the cornerstone of clinical risk stratification in the contemporary ICI-TKI era, its ability to accurately predict prognosis remains suboptimal (4, 6–8). Furthermore, the IMDC model relies primarily on host physiological parameters and basic blood counts, failing to incorporate the complex immunological interactions within the tumor microenvironment (TME). While systemic inflammatory markers such as the neutrophil-to-lymphocyte ratio (NLR) and systemic immune-inflammation index (SII) have shown prognostic value, the role of the humoral immune system remains relatively under-explored (9–12).

Traditionally, serum IgG has been utilized as a functional readout of the humoral immune system, where its levels reflect the host’s systemic immune competence and chronic inflammatory status (13, 14). However, a paradigm shift in cancer biology has occurred with the discovery of “cancer-derived IgG” (CIgG), which is produced directly by malignant epithelial cells rather than B-lineage cells (15). CIgG has been shown to exhibit potent pro-tumorigenic effects by activating oncogenic signaling pathways, such as MEK/ERK and FAK-Src, while simultaneously facilitating immune evasion (15, 16). In clear cell renal cell carcinoma (ccRCC), high tissue expression of IgG is closely linked to advanced clinical stage, poor histological differentiation, and diminished prognosis (17, 18).

To date, the prognostic value of baseline (pretreatment) serum IgG in mRCC patients receiving first-line ICI–TKI combinations has not been established. In this study, we aimed to evaluate whether baseline serum IgG levels provide independent prognostic information in mRCC patients treated with modern first-line targeted immunotherapy, potentially complementing the IMDC framework for more personalized clinical management.

## 2 Methods

### 2.1 Study design and patient population

This retrospective cohort study included patients with mRCC who received first-line immune checkpoint inhibitor plus tyrosine kinase inhibitor (ICI–TKI) combination therapy in the National Cancer Center/National Clinical Research Center for Cancer/Cancer Hospital, Chinese Academy of Medical Sciences and Peking Union Medical College between May 2019 and April 2026. The study was conducted in accordance with the Strengthening the Reporting of Observational Studies in Epidemiology (STROBE) guidelines.

Among 405 patients with metastatic renal cell carcinoma (mRCC) who received first-line ICI-TKI therapy between May 2019 and April 2026, 252 were excluded (171 with history of other malignancies or previous systemic antitumor therapy, 28 with missing baseline serum IgG measurements, and 53 with missing key clinicopathological or follow-up data), leaving 153 patients for the final analysis.

Eligible patients met the following inclusion criteria: (1) age ≥18 years; (2) newly diagnosed with primary renal cell carcinoma and metastatic sites; and (3) receipt of first-line ICI–TKI therapy. Patients were excluded if they had a history of other malignant tumors or previous systemic antitumor therapy, lacked pretreatment serum immunoglobulin G measurements, or had missing key clinicopathological or follow-up data. Of the 405 patients screened during the study period, 252 were excluded (171 with a history of other malignancies or previous systemic therapy, 28 lacking baseline serum IgG measurements, and 53 with missing key clinicopathological or follow-up data), leaving 153 patients for the final analysis. The patient selection process is presented in a STROBE flowchart (**Figure 1**).

**Figure 1:**
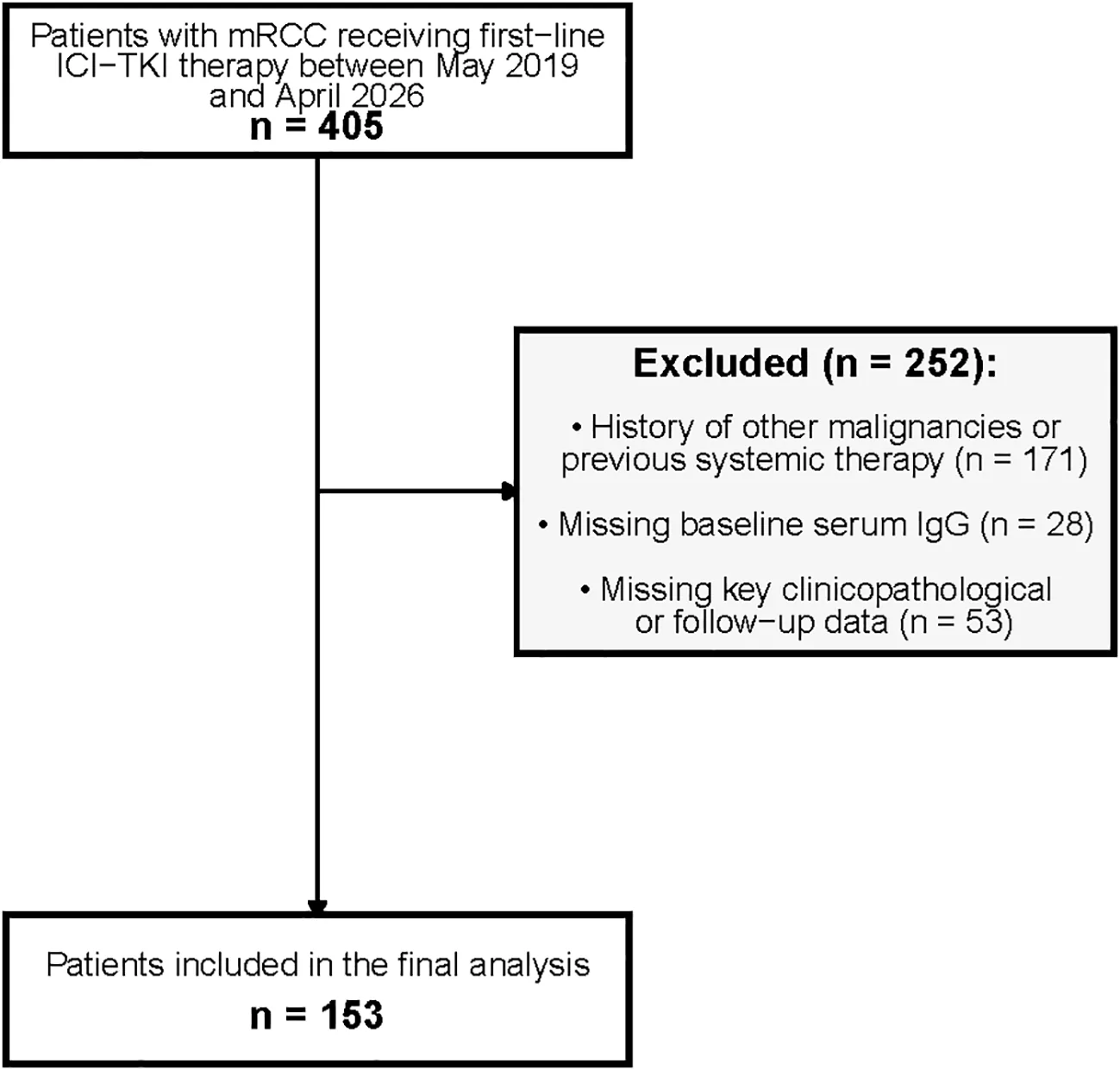
STROBE flowchart of patient selection.

The study protocol was approved by the Ethics Committee of the National Cancer Center/National Clinical Research Center for Cancer/Cancer Hospital, Chinese Academy of Medical Sciences and Peking Union Medical College (approval number: 20/245-2441). Given the retrospective nature of the study, the requirement for informed consent was waived.

### 2.2 Data collection

Clinical and pathological data were extracted from electronic medical records, including age, sex, histological subtype, IMDC risk classification, metastatic burden, treatment regimen, and survival outcomes. Baseline serum immunoglobulin levels were obtained from routine laboratory assessments performed before initiation of first-line ICI–TKI therapy using standard institutional assays. Baseline inflammatory markers, including the neutrophil-to-lymphocyte ratio (NLR) and C-reactive protein (CRP), were also obtained from routine pretreatment blood tests.

### 2.3 Definition of baseline serum IgG status

Baseline serum IgG levels were assessed in our institutional clinical laboratory, with a reference range of 8.6–17.4 g/L. To further assess the robustness of this threshold, outcome-oriented cut-off analysis was performed using maximally selected rank statistics implemented in the surv_cutpoint function of the *survminer* R package. The optimal cut-off identified by survival-based analysis was 17.56 g/L, which was highly consistent with the institutional upper reference limit. Therefore, 17.4 g/L was used to define elevated baseline serum IgG in all subsequent analyses. Patients were stratified into two groups based on the upper limit of the reference interval: those with IgG levels >17.4 g/L were classified as elevated IgG, whereas all remaining patients (≤17.4 g/L, including those below the lower reference limit) were categorized as normal IgG. The normal group included 8 patients (5.2%) with IgG below the institutional lower reference limit (8.6 g/L); a three-group sensitivity analysis was performed to evaluate this subgroup separately.

### 2.4 Follow-up and response assessment

Patients were regularly followed after initiation of first-line ICI–TKI therapy. During the treatment period, physical examinations, laboratory tests (including complete blood count, liver and renal function, and electrolytes), and adverse event assessment were performed at each treatment visit, typically every 2–3 weeks.

Tumor imaging with contrast-enhanced computed tomography (CT) or magnetic resonance imaging (MRI) of the chest, abdomen, and pelvis was performed every 1–3 months during treatment, or sooner if clinically indicated (e.g., new symptoms). Treatment response was assessed by the treating physicians according to the Response Evaluation Criteria in Solid Tumors (RECIST) version 1.1. Disease progression was defined based on radiographic evidence of new lesions or significant increase in size of known lesions, confirmed by subsequent imaging or clinical deterioration.

### 2.5 Study endpoints

Progression-free survival (PFS) was defined as the time from initiation of first-line ICI–TKI therapy to disease progression or death from any cause, whichever occurred first. Overall survival (OS) was defined as the time from treatment initiation to death from any cause. Patients without documented events were censored at the date of last follow-up.

### 2.6 Statistical analysis

Continuous variables are presented as median and interquartile range (IQR), whereas categorical variables are summarized as number and percentage. Comparisons between groups were performed using the Mann–Whitney U test for continuous variables and the χ² test or Fisher’s exact test for categorical variables, as appropriate.

Survival curves were estimated using the Kaplan–Meier method and compared using the log-rank test. Univariable and multivariable Cox proportional hazards regression models were used to evaluate prognostic factors associated with overall survival. Variables for the multivariable model were prespecified *a priori* based on clinical relevance and biological plausibility and included age, sex, IMDC risk group, and baseline serum IgG group. No data-driven variable selection was performed. Hazard ratios (HRs) and 95% confidence intervals (CIs) were calculated.

Time-dependent receiver operating characteristic (ROC) curves and Harrell’s concordance index (C-index) were used to assess prognostic discrimination. Internal validation was performed using bootstrap resampling (1000 iterations) to estimate optimism-corrected model performance and calibration. Calibration plots were generated to evaluate agreement between predicted and observed survival probabilities at 1, 2, and 3 years. The proportional hazards assumption was assessed using Schoenfeld residuals.

A prognostic nomogram for individualized prediction of 1-, 2-, and 3-year overall survival was developed based on the final multivariable Cox regression model.

To evaluate whether the dichotomous IgG classification adequately captured the relationship between IgG and survival, we performed supplementary analyses using restricted cubic splines (RCS) with 3 knots placed at the 10th, 50th, and 90th percentiles of IgG. The nonlinear component was tested using the Wald test. Additionally, a sensitivity analysis was performed by categorizing patients into three groups based on institutional reference ranges: low (<8.6 g/L), normal (8.6–17.4 g/L), and high (>17.4 g/L). Kaplan–Meier curves and log-rank tests were used to compare survival across the three groups, and Cox proportional hazards models were used to estimate hazard ratios with the normal IgG group as reference.

To evaluate whether the prognostic value of IgG was independent of systemic inflammation, we constructed nested Cox proportional hazards models by incrementally adding established inflammatory markers (NLR and CRP) to the base model (Model 1: IgG group + age + sex + IMDC risk group). Model 2 added NLR as a continuous variable, and Model 3 further added CRP as a continuous variable. The change in the hazard ratio for elevated IgG across models was examined to assess the degree to which inflammation confounded the IgG–survival association. All statistical analyses were performed using R software (version 4.6.0; R Foundation for Statistical Computing, Vienna, Austria). A two-sided P value <0.05 was considered statistically significant.

## 3 Results

### 3.1 Baseline clinicopathological characteristics of the patients

A total of 153 patients with mRCC who received first-line ICI-TKI therapy between May 2019 and April 2026 were included in the final analysis. Among first-line ICI–TKI regimens in patients with mRCC, axitinib plus pembrolizumab was the most commonly administered (56.9%), followed by lenvatinib plus pembrolizumab (20.3%) and axitinib plus toripalimab (11.7%); other regimens accounted for 11.1%. In terms of histological subtypes among patients with mRCC, ccRCC was predominant (90.2%), followed by papillary renal cell carcinoma (6.5%) and chromophobe renal cell carcinoma (1.3%), while other histological subtypes accounted for 2.0%.

At the time of data cutoff for the final analysis, 44 deaths had occurred. The median follow-up duration, estimated using the reverse Kaplan–Meier method, was 29.0 months (95% confidence interval [CI], 26.0–39.0). The median OS was 48.3 months (95% CI, 44.0–not reached), while the median PFS was 17.2 months (95% CI, 15.0–23.2). The estimated 1-, 2-, and 3-year OS rates were 93.2%, 81.8%, and 63.4%, respectively, whereas the estimated 1- and 2-year progression-free survival rates were 65.9% and 40.2%, respectively.

Demographic and clinical characteristics of the 153 patients, stratified by baseline IgG level (normal IgG, n=126; elevated IgG, n=27), are detailed in **Table 1**. The median age of the overall cohort was 59 years (IQR, 54–65), with no significant intergroup difference (P = 0.872). Most patients were male (77.8%, 119/153); the proportion was higher in the normal IgG group (81.0%) than the elevated IgG group (63.0%), without statistical significance (P = 0.124). For tumor characteristics, 50.3% (77/153) of patients had T1–2 stage and 49.7% (76/153) had T3–4 stage, with no significant intergroup difference in T stage distribution (P = 0.103). N1 stage was present in 18.3% (28/153), and 36.6% (56/153) had ≥2 metastatic sites, with no significant intergroup differences (P = 0.429 and P = 0.959, respectively). The IMDC risk group distribution differed significantly between groups (P = 0.022): the normal IgG group had a higher proportion of favorable IMDC risk (27.8% vs. 3.7%), while the elevated IgG group had higher proportions of intermediate (70.4% vs. 57.1%) and poor (25.9% vs. 15.1%) risk. As expected, median baseline IgG was significantly higher in the elevated IgG group (20.43 g/L, IQR 18.00–23.80) than the normal IgG group (12.50 g/L, IQR 10.93–14.33; P<0.001).

**Table 1:** Baseline clinicopathological characteristics of the 153 patients with mRCC stratified by baseline serum IgG levels.

Variable	Overall (n=153)	Normal IgG (n=126)	Elevated IgG (n=27)	P value
Age, years (IQR)	59 (54–65)	59.5 (54–65)	59 (55.5–65)	0.872
Male, n (%)	119 (77.8)	102 (81.0)	17 (63.0)	0.124
T stage, n (%)				0.103
T1–2	77 (50.3)	67 (53.2)	10 (37.0)	
T3–4	76 (49.7)	59 (46.8)	17 (63.0)	
N1 stage, n (%)	28 (18.3)	25 (19.8)	3 (11.1)	0.429
≥2 metastatic sites, n (%)	56 (36.6)	46 (36.5)	10 (37.0)	0.959
IMDC risk group, n (%)				0.022
Favorable	36 (23.5)	35 (27.8)	1 (3.7)	
Intermediate	91 (59.5)	72 (57.1)	19 (70.4)	
Poor	26 (17.0)	19 (15.1)	7 (25.9)	
Baseline IgG, g/L (IQR)	13.29 (11.30–15.90)	12.50 (10.93–14.33)	20.43 (18.00–23.80)	–

Continuous variables are presented as median (interquartile range [IQR]) and were compared using the Mann–Whitney U test. Categorical variables are presented as number (%) and were compared using the χ² test or Fisher’s exact test, as appropriate. Baseline serum IgG was used to define the study groups; therefore, no statistical comparison was performed.

IMDC, International Metastatic Renal Cell Carcinoma Database Consortium; IgG, immunoglobulin G; IQR, interquartile range.

### 3.2 Potential prognostic value of baseline serum IgG

Elevated baseline serum IgG levels were significantly associated with poorer progression-free survival compared with normal IgG levels (log-rank P = 0.049; **Figure 2A**). Similarly, patients with elevated serum IgG levels exhibited significantly worse overall survival than those with normal IgG levels (log-rank P < 0.001; **Figure 2B**).

**Figure 2:**
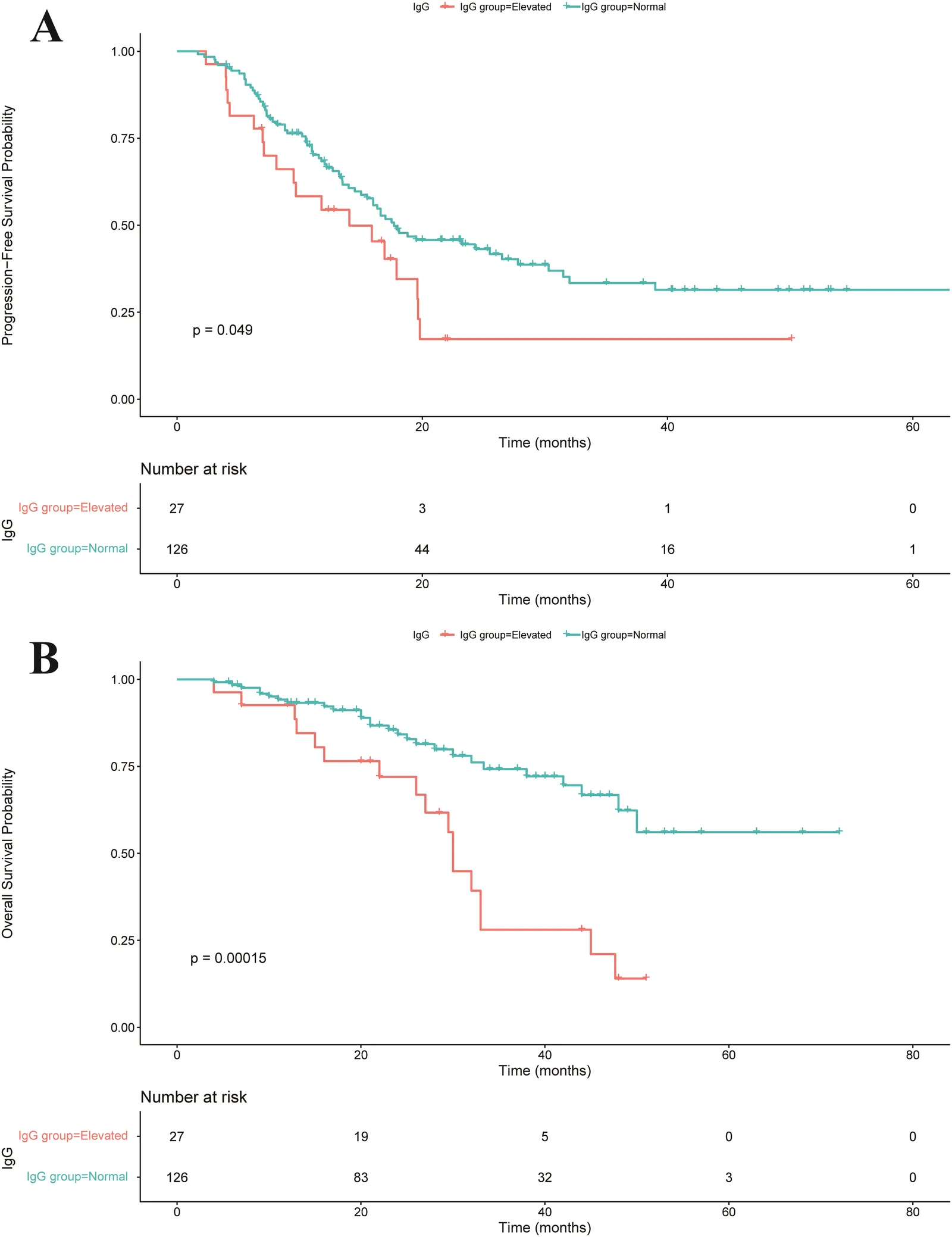
Kaplan–Meier survival curves according to baseline serum IgG levels. **(A)** Progression-free survival (PFS). **(B)** Overall survival (OS). Patients were stratified into normal and elevated IgG groups using the institutional upper normal reference limit of 17.4 g/L. Survival differences were compared using the log-rank test.

PFS was further evaluated using Cox proportional hazards regression models. In univariable analysis, sex, age, T stage, and the number of metastatic sites were not significantly associated with PFS (all P > 0.05). Patients with N1 disease tended to have shorter PFS than those with N0 disease, although the association did not reach statistical significance (HR = 1.62, 95% CI 0.99–2.65, P = 0.054). Among IMDC risk groups, poor-risk patients experienced significantly worse PFS compared with favorable-risk patients (HR = 2.06, 95% CI 1.07–3.97, P = 0.031), whereas no significant difference was observed for the intermediate-risk group. Elevated baseline serum IgG was associated with a tendency toward inferior PFS in univariable analysis (HR = 1.66, 95% CI 1.00–2.77, P = 0.052).

After adjustment for age, sex, and IMDC risk category in the multivariable model, elevated serum IgG was no longer significantly associated with PFS (HR = 1.60, 95% CI 0.93–2.77, P = 0.090). Likewise, the adverse effect of poor IMDC risk on PFS was attenuated and failed to maintain statistical significance in multivariable analysis (HR = 1.91, 95% CI 0.98–3.74, P = 0.058) (**Table 2**).

**Table 2:** Univariable and multivariable Cox proportional hazards regression analyses for progression-free survival.

	Univariate			Multivariate		
Variable	HR	95% CI	*P*-values	HR	95% CI	*P*-value
Sex						
Female	Reference			Reference		
Male	1.08	0.65-1.82	0.764	1.30	0.75-2.24	0.344
Age	0.99	0.96-1.01	0.264	0.99	0.97-1.01	0.277
Tprimary						
1	Reference					
2	1.31	0.72-2.39	0.379			
3	1.47	0.87-2.48	0.148			
4	1.69	0.72-3.95	0.228			
N						
0	Reference					
1	1.62	0.99-2.65	0.054			
Number of metastatic sites						
1	Reference					
2	1.19	0.75-1.88	0.468			
3	1.59	0.65-3.99	0.321			
4	0.48	0.07-3.51	0.473			
IMDC						
Favorable	Reference			Reference		
Intermediate	1.18	0.69-2.01	0.553	1.10	0.64-1.91	0.723
Poor	2.06	1.07-3.97	0.031	1.91	0.98-3.74	0.058
IgG group						
Normal IgG	Reference			Reference		
Elevated IgG	1.66	1.00-2.77	0.052	1.60	0.93-2.77	0.090

Hazard ratios (HRs) and 95% confidence intervals (CIs) were estimated using Cox proportional hazards regression models. Variables included in the multivariable analysis were age, sex, IMDC risk group, and baseline serum IgG group.

CI, confidence interval; HR, hazard ratio; IMDC, International Metastatic Renal Cell Carcinoma Database Consortium; IgG, immunoglobulin G.

In univariable Cox proportional hazards regression analysis, male sex, age, T stage, N stage, and the number of metastatic sites were not significantly associated with OS (all P > 0.05). Within the IMDC risk classification, patients with poor-risk disease demonstrated a significantly increased risk of death compared with those with favorable-risk disease (HR = 3.78, 95% CI 1.33–10.70, P = 0.013), while intermediate-risk disease showed a non-significant trend toward worse OS (HR = 1.91, 95% CI 0.73–4.97, P = 0.184). Importantly, elevated baseline serum IgG was significantly associated with inferior OS in univariable analysis (HR = 3.09, 95% CI 1.68–5.67, P < 0.001).

In multivariable Cox regression analysis adjusting for age, sex, and IMDC risk group, elevated baseline serum IgG remained an independent prognostic factor for worse OS (HR = 2.55, 95% CI 1.30–5.00, P = 0.006). Poor IMDC risk showed a trend toward inferior survival but did not reach statistical significance in the multivariable model (HR = 2.86, 95% CI 0.95–8.37, P = 0.062) (**Table 3**).

**Table 3:** Univariable and multivariable Cox proportional hazards regression analyses for overall survival.

	Univariate			Multivariate		
Variable	HR	95% CI	*P*-value	HR	95% CI	*P*-value
Sex						
Female	Reference			Reference		
Male	0.62	0.32-1.20	0.152	0.78	0.37-1.66	0.518
Age	0.98	0.96-1.01	0.305	1.00	0.97-1.03	0.866
Tprimary						
1	Reference					
2	0.75	0.30-1.84	0.526			
3	1.26	0.61-2.60	0.529			
4	2.13	0.75-6.08	0.157			
N						
0	Reference					
1	1.50	0.74-3.03	0.265			
Number of metastatic sites						
1	Reference					
2	1.31	0.68-2.53	0.423			
3	2.13	0.50-9.12	0.306			
4	1.45	0.20-10.8	0.717			
IMDC						
Favorable	Reference			Reference		
Intermediate	1.91	0.73-4.97	0.184	1.44	0.53-3.91	0.477
Poor	3.78	1.33-10.7	0.013	2.86	0.95-8.37	0.062
IgG group						
Normal IgG	Reference			Reference		
Elevated IgG	3.09	1.68-5.67	<0.001	2.55	1.30-5.00	0.006

Hazard ratios (HRs) and 95% confidence intervals (CIs) were estimated using Cox proportional hazards regression models. Variables included in the multivariable analysis were age, sex, IMDC risk group, and baseline serum IgG group.

CI, confidence interval; HR, hazard ratio; IMDC, International Metastatic Renal Cell Carcinoma Database Consortium; IgG, immunoglobulin G.

Sensitivity analyses restricted to patients with clear cell renal cell carcinoma (n = 138) and to those receiving pembrolizumab plus axitinib (n = 87) yielded consistent results, with high IgG remaining significantly associated with poorer OS in both subgroups (HR = 2.51, P = 0.007 and HR = 2.70, P = 0.006, respectively) and a consistent trend for PFS (HR = 1.60, P = 0.090 and HR = 1.66, P = 0.091, respectively) (**Supplementary Table 1**).

### 3.3 Restricted cubic spline and three-group sensitivity analysis

RCS analysis revealed a significant nonlinear relationship between baseline IgG and both OS(nonlinear P = 0.005) and PFS (nonlinear P = 0.015). The dose–response curves showed a U-shaped pattern, with elevated hazard ratios at both low and high IgG levels and the lowest risk observed at approximately 12–14 g/L (**Supplementary Figure 1**).

When patients were stratified into three groups, 8 (5.2%) had low IgG, 118 (77.1%) had normal IgG, and 27 (17.6%) had high IgG. The log-rank test showed significant differences among the three groups for both OS (P < 0.001) and PFS (P = 0.012) (**Supplementary Figure 2**).

On multivariate Cox analysis, high IgG (>17.4 g/L) remained significantly associated with worse OS compared to normal IgG (HR = 2.69, 95% CI: 1.37–5.29, P = 0.004), consistent with the primary dichotomous analysis. For PFS, high IgG showed a borderline association (HR = 1.66, 95% CI: 0.96–2.87, P = 0.072), with the hazard ratio direction consistent with the OS finding. Low IgG (<8.6 g/L) was associated with worse OS (HR = 3.55, 95% CI: 1.00–12.58, P = 0.050) and PFS (HR = 2.53, 95% CI: 1.03–6.18, P = 0.042), but the wide confidence intervals reflected the limited sample size (n = 8) in this group (**Supplementary Table 2**).

### 3.4 Incremental prognostic value of IgG beyond inflammatory markers

To investigate whether the prognostic association of baseline IgG was independent of systemic inflammation, we performed nested Cox regression analyses by sequentially adding NLR and CRP to the base model (age, sex, IMDC risk group, and IgG group).

For OS, elevated IgG remained significantly associated with worse survival across all three models. The hazard ratio for high IgG was 2.51 (95% CI: 1.29–4.88, P = 0.007) in the base model, 2.54 (95% CI: 1.31–4.95, P = 0.006) after adding NLR, and 2.40 (95% CI: 1.22–4.72, P = 0.011) after further adding CRP. The minimal attenuation of the HR (approximately 4% from Model 1 to Model 3) indicated that the prognostic effect of IgG was largely independent of these inflammatory markers (**Supplementary Table 3**).

For PFS, elevated IgG showed a consistent trend toward worse progression-free survival across nested models, although the association did not reach conventional statistical significance. The HR was 1.60 (95% CI: 0.93–2.77, P = 0.090) in the base model, 1.68 (95% CI: 0.97–2.91, P = 0.065) after adding NLR, and 1.60 (95% CI: 0.92–2.79, P = 0.099) after adding CRP. The direction and magnitude of the effect remained stable, supporting independence from inflammatory markers.

In the fully adjusted model (Model 3), NLR showed a borderline association with PFS (HR per unit = 1.13, 95% CI: 1.00–1.28, P = 0.060) but not OS (P = 0.338), while CRP was not significantly associated with either outcome (both P > 0.3).

### 3.5 Prognostic performance and model validation

The time-dependent discriminative ability of the prognostic models was further evaluated using receiver operating characteristic (ROC) analysis at 1-, 2-, and 3-year time points. Compared with the IMDC-only model, the incorporation of baseline serum IgG demonstrated variable improvements in predictive performance across different time horizons. At 1 year, the area under the curve (AUC) was comparable between the two models (IMDC vs IMDC + IgG: 0.719 vs 0.718; ΔAUC = −0.001, bootstrap 95% CI: −0.095 to 0.046). However, a clear improvement was observed at later time points, with higher AUC values for the combined model at 2 years (0.657 vs 0.694; ΔAUC = 0.037, bootstrap 95% CI: −0.040 to 0.074) and 3 years (0.672 vs 0.745; ΔAUC = 0.073, bootstrap 95% CI: 0.012–0.132), suggesting that baseline IgG contributes more substantially to long-term prognostic stratification (**Figure 3**).

**Figure 3:**
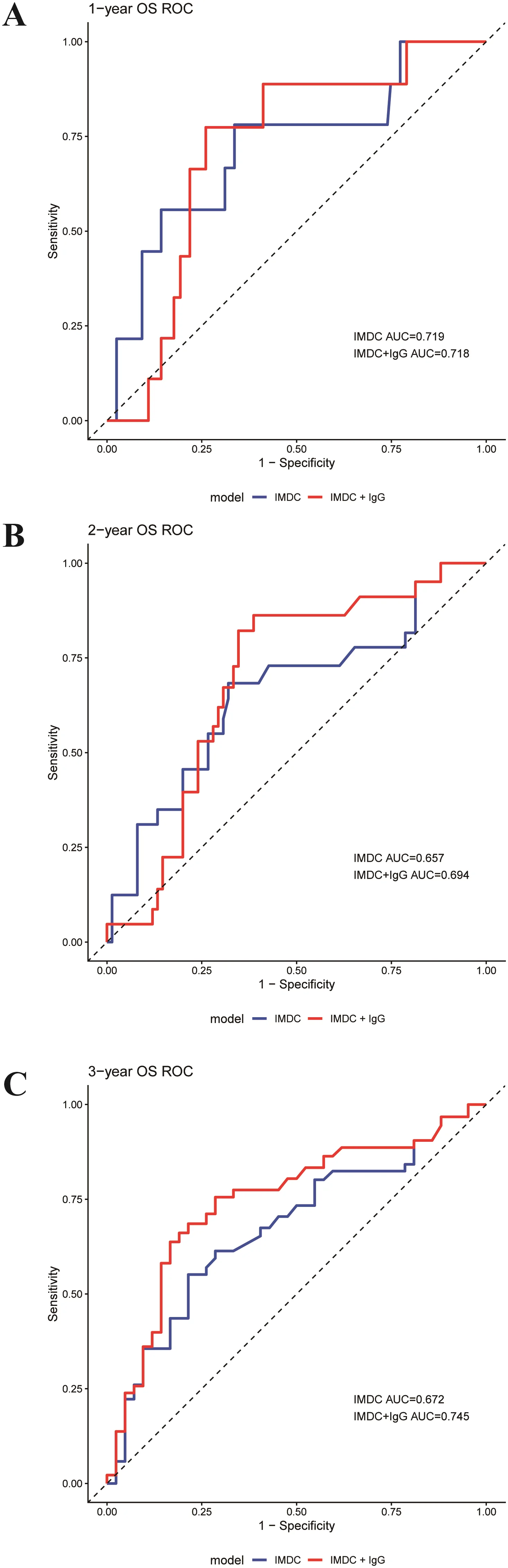
Time-dependent ROC curves for overall survival prediction. Time-dependent receiver operating characteristic (ROC) curves comparing the prognostic performance of the IMDC model and the IMDC + baseline IgG model at 1-, 2-, and 3-year time points. **(A)** 1-year ROC analysis (AUC: 0.719 vs 0.718). **(B)** 2-year ROC analysis (AUC: 0.657 vs 0.694). **(C)** 3-year ROC analysis (AUC: 0.672 vs 0.745).

The discriminatory performance of the prognostic models was evaluated using Harrell’s concordance index (C-index).Compared with the IMDC-only model, incorporation of baseline serum IgG improved the discriminative ability for overall survival prediction (C-index: 0.689, 95% CI 0.655–0.723 vs 0.645, 95% CI 0.610–0.680). The absolute increase in C-index was 0.044 (bootstrap 95% CI: −0.005 to 0.082), and the direction of improvement was consistent across time-dependent ROC analyses, with the greatest increment observed at 3 years (ΔAUC = 0.073, bootstrap 95% CI: 0.012–0.132), suggesting that baseline serum IgG contributes incremental prognostic value, particularly for longer-term outcomes. (**Supplementary Table 4**).

The multivariable Cox regression model incorporating IMDC risk group, age, sex, and baseline serum IgG demonstrated an apparent Harrell’s C-index of 0.712. Following bootstrap internal validation with 1000 resamples, the optimism-corrected C-index was 0.676. The calibration slope was 0.79, indicating limited overfitting and acceptable model performance (**Supplementary Table 5**).

Model calibration for 1-, 2-, and 3-year OS was evaluated using bootstrap resampling with 1,000 repetitions. The 3-year OS prediction showed excellent agreement with actual observations, yielding a low mean absolute error of 0.069. Although the 1- and 2-year curves displayed marginally conservative tendencies (mean absolute errors: 0.221 and 0.128, respectively), the model demonstrated high reliability and precise calibration for long-term (3-year) survival estimation (**Supplementary Figure 3**).

The proportional hazards assumption was assessed using Schoenfeld residuals. Although the global test reached borderline significance for OS (P = 0.032), individual covariate tests showed no significant violation for baseline serum IgG group (P = 0.319 for OS; P = 0.730 for PFS). The global test significance was primarily driven by IMDC risk group (P = 0.050). To formally evaluate whether the IgG effect varied over time, a time-dependent interaction test was performed by adding an IgG × log(time) interaction term to the Cox model. The interaction was non-significant for both OS (P = 0.404) and PFS (P = 0.996), confirming that the prognostic effect of IgG did not change over time. Additionally, a stratified Cox model with IMDC as the strata variable was fitted as an alternative modeling approach. In this model, the global Schoenfeld test was no longer significant (P = 0.060 for OS; P = 0.400 for PFS), and elevated IgG remained significantly associated with worse OS (HR = 2.40, 95% CI: 1.22–4.73, P = 0.012), consistent with the primary analysis (**Supplementary Table 6**, **Supplementary Figure 4**).

### 3.6 Nomogram for individualized overall survival prediction

An exploratory prognostic nomogram was constructed based on the final multivariable Cox regression model incorporating IMDC risk group, age, sex, and baseline serum IgG group. The nomogram provided individualized predictions of 1-, 2-, and 3-year overall survival probabilities (**Figure 4**) but requires external validation before clinical application.

**Figure 4:**
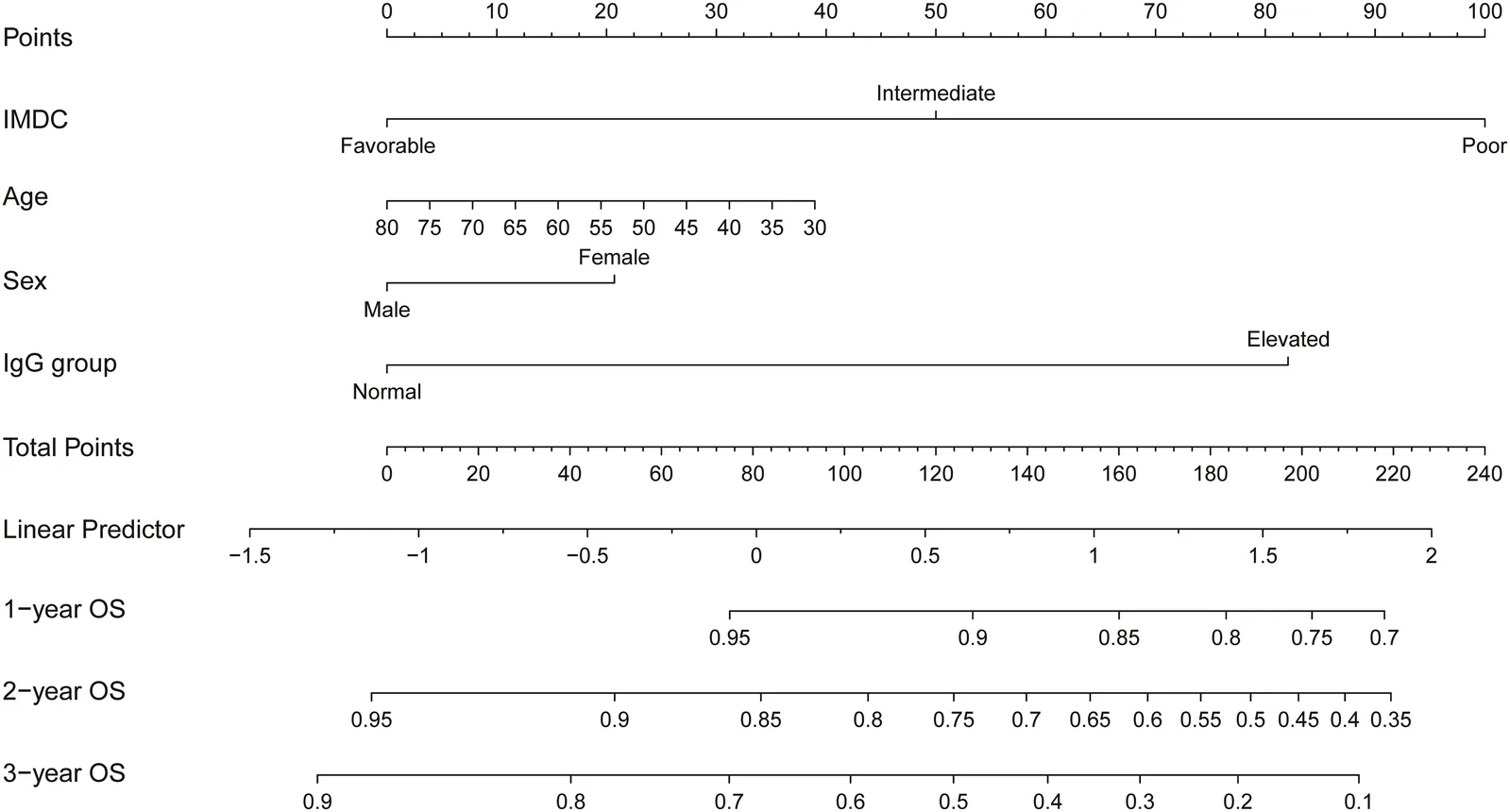
Nomogram for individualized prediction of 1-, 2-, and 3-year overall survival. Nomogram developed from the final multivariable Cox proportional hazards regression model incorporating age, sex, IMDC risk group, and baseline serum IgG group. IMDC, International Metastatic Renal Cell Carcinoma Database Consortium; IgG, immunoglobulin G.

## 4 Discussion

The primary finding of this study is that baseline serum IgG level serves as an independent prognostic factor for OS in patients with mRCC receiving first-line ICI-TKI combination therapy. Specifically, patients with elevated baseline IgG (>17.4 g/L) exhibited significantly shorter OS compared to those with normal levels, a relationship that remained robust after adjusting for age, sex, and the IMDC risk group (HR = 2.55, 95% CI: 1.30–5.00, P = 0.006). In contrast, although elevated baseline IgG was associated with shorter PFS in unadjusted Kaplan–Meier analysis, this association was not confirmed in multivariable Cox regression, suggesting that its effect on early disease progression may be partially confounded by established clinical risk factors. This discovery highlights the critical role of the baseline humoral immune environment in determining the success of modern immunotherapy-based regimens and suggests that IgG captures biological nuances of the tumor-host interaction that other clinical parameters do not.

A subtle discrepancy between our findings and previous research from our institution warrants detailed discussion. A previous study have reported that baseline IgG levels were not significantly associated with OS or objective response rates (ORR) in their analysis of mRCC cohorts (19). However, we believe this discrepancy stems from fundamental differences in cohort composition and therapeutic modes. This study included a heterogeneous cohort of patients who received TKI monotherapy(69%)as their first-line treatment (19). TKI monotherapy primarily functions by inhibiting angiogenesis and tumor cell proliferation through the VEGF/VEGFR pathway, mechanisms that are relatively independent of the host’s baseline systemic immune status. In such mixed cohorts, the predictive signal of baseline IgG—which is inherently an immunological marker—was diluted by the presence of many patients whose outcomes were driven by non-immunological drug mechanisms. In contrast, our current study focused exclusively on a cohort receiving first-line ICI-TKI therapy. The efficacy of the ICI component in this regimen is fundamentally reliant on the restoration of the host’s anti-tumor immune response. By focusing on a select cohort whose therapeutic outcomes are more heavily contingent on immune activation, we successfully unveiled the prognostic value of baseline IgG—a clinical signal that was likely obscured by the predominant anti-angiogenic mechanisms of TKI monotherapy in previous studies.

To address the concern that dichotomizing IgG may oversimplify its prognostic relationship, we performed RCS analysis, which confirmed a significant nonlinear (U-shaped) association between IgG and survival. However, the elevated risk at low IgG levels was based on only 8 patients (5.2% of the cohort), precluding reliable subgroup estimation. The three-group sensitivity analysis confirmed that the prognostic effect of high IgG on OS remained robust (HR = 2.69, P = 0.004), supporting the validity of using 17.4 g/L as the cutoff in the primary analysis. The borderline PFS finding (P = 0.072) in the three-group analysis is likely attributable to reduced statistical power from subgroup splitting rather than a true loss of effect, as the hazard ratio remained consistent with the primary analysis.

Circulating IgG levels in patients with mRCC may represent a complex biological integration of both tumor-related and host-derived processes. On one hand, they may reflect the baseline status of the adaptive immune system; on the other, they potentially represent the secretory output of aggressive, CIgG-producing tumor clones. Although prior research has primarily focused on the prognostic significance of IgG within the TME, the clinical relevance of circulating serum IgG in the era of ICI-TKI combination therapy remains largely unexplored. From this perspective, total serum IgG may serve as a composite biomarker integrating both tumor intrinsic aggressiveness and host immune status.

To further elucidate the potential biological basis of this observation, the mechanisms underlying elevated serum IgG can be considered from both tumor- and host-related perspectives. However, it is important to note that total serum IgG measurements cannot distinguish host-derived from cancer-derived IgG, nor can they distinguish sialylated isoforms or specific subclasses such as IgG4. The following discussion therefore represents a hypothetical framework to guide future investigation, rather than a definitive mechanistic explanation for our findings. From the tumor perspective, IgG has recently been identified as being expressed and secreted by cancer cells themselves, a phenomenon termed CIgG (15, 20). Extensive work has demonstrated that sialylated cancer-derived IgG (SIgG) is overexpressed in renal cell carcinoma and acts as a potent promoter of immune escape (24). SIgG can directly inhibit the proliferation of effector CD4+ and CD8+ T cells by binding to sialic acid-binding immunoglobulin-type lectins (Siglecs) on their surface, thereby creating an immunosuppressive microenvironment that blunts the efficacy of PD-1/PD-L1 blockade (18, 21–23). If a substantial fraction of the elevated serum IgG in these patients were tumor-derived and sialylated, it could potentially contribute to an immunosuppressive milieu; however, direct evidence for this in the present cohort is lacking, and this remains a hypothesis requiring dedicated validation. From the host perspective, the contribution of host-derived B cells and their immunoglobulins must be interpreted with biological nuance. While localized B-cell infiltration and mature tertiary lymphoid structures (TLS) within the TME are generally associated with favorable responses to ICIs, a non-specific elevation in total circulating IgG represents a completely different pathological process (19, 24–26). Clinically, elevated circulating IgG is tightly coupled with systemic inflammatory and hypercoagulable states, showing strong positive correlations with classical markers such as C-reactive protein (CRP), fibrinogen (FIB), and the NLR (27). This indicates that high baseline IgG also serves as a surrogate marker for a broader systemic inflammatory response (SIR) and coagulation activation—both of which are well-established hallmarks of aggressive disease and immunotherapy resistance (28–32). Importantly, nested Cox regression analyses demonstrated that the prognostic value of elevated baseline IgG for OS was largely independent of established systemic inflammatory markers, including NLR and CRP. The hazard ratio for high IgG decreased by only approximately 4% after adjustment for both NLR and CRP (from HR = 2.51 to HR = 2.40) and remained statistically significant (P = 0.011). This finding suggests that the prognostic information captured by serum IgG goes beyond what is reflected by conventional inflammatory markers, supporting the notion that IgG may encompass additional biological dimensions such as cancer-derived immunoglobulin production and humoral immune dysregulation within the tumor microenvironment.

Taken together, while both tumor-derived SIgG and host-derived chronic inflammation could theoretically contribute to immune evasion in the context of elevated IgG, further studies with subclass-specific and glycoform-specific measurements are needed to determine which of these pathways predominates in mRCC patients receiving ICI-TKI therapy. Regardless of the precise underlying mechanism, however, the consistent prognostic association we observed suggests that total serum IgG captures clinically relevant biological information about the tumor-host interaction. Consequently, as a non-invasive, easily accessible circulating biomarker, baseline serum IgG holds substantial clinical potential to integrate these dual pathological dimensions and provide additional prognostic information for patients with mRCC receiving ICI-TKI therapy.

From a clinical perspective, the incremental benefit of incorporating IgG into existing models is substantial. While the IMDC risk model remains the well-established standard for mRCC prognosis, its performance in the era of combination immunotherapy has shown limitations (33, 34). In our cohort, adding baseline IgG status to the IMDC model improved the C-index from 0.645 to 0.689. This improvement suggests that IgG captures immunological nuances of the tumor-host interaction that traditional laboratory parameters like hemoglobin or calcium do not. The nomogram developed in this study provides clinicians with a practical tool to refine individual risk assessments and potentially tailor follow-up or treatment strategies for patients in the intermediate-risk category who may have an unexpectedly high risk of death due to elevated IgG levels.

Notably, the improvement in predictive performance achieved by adding baseline serum IgG was more pronounced for 3-year OS (ΔAUC = 0.073) than for 1-year OS (ΔAUC = −0.001). This time-dependent difference can be attributed to several factors. First, the 1-year OS rate was 93.2%, meaning that very few events (approximately 10 deaths) had occurred within the first year, limiting the discriminative capacity of any model at this early timepoint. By 3 years, the event rate had increased substantially (3-year OS rate, 63.4%), providing a greater number of outcomes for model discrimination. Second, the predictive accuracy of the IMDC classification itself has been shown to decline over time, with concordance indices dropping below 0.70 beyond 2 years, thereby creating more room for additional biomarkers to contribute at longer follow-up durations (6). Third, from a biological perspective, short-term outcomes are predominantly driven by tumor burden and initial treatment sensitivity—factors already captured by the IMDC classification—whereas long-term outcomes are increasingly influenced by the host immune microenvironment and sustained immune competence, which serum IgG may reflect.

Despite these findings, several limitations should be noted. This was a single-center retrospective study with a relatively limited sample size, which may introduce selection bias. Second, this was a single-arm study of ICI-TKI therapy without a comparator arm; therefore, baseline IgG should be interpreted as a prognostic biomarker rather than a predictive biomarker of ICI efficacy. With only 44 death events, the prognostic model showed moderate performance with some overfitting (optimism-corrected C-index = 0.676, calibration slope = 0.79), and the nomogram should be interpreted as exploratory pending external validation. Although bootstrap internal validation and calibration analysis were performed, external validation in independent multicenter cohorts is essential before baseline serum IgG or the nomogram can be incorporated into routine clinical practice. Although we adjusted for established inflammatory markers and the clinical eligibility criteria for ICI-TKI therapy inherently excluded patients with severe comorbidities, the possibility that undiagnosed conditions may have influenced serum IgG levels cannot be entirely excluded. Additionally, our analysis measured total serum IgG rather than specific subclasses; since research indicates that IgG4 may specifically interfere with ICI efficacy through Fc-Fc interactions, future studies should focus on immunoglobulin subclass profiling to further refine the predictive value (35–38). Finally, while we have established the importance of baseline values, integrating baseline levels with dynamic changes during treatments suggested by a previous study identifying the value of 3-month IgG changes—may provide even greater prognostic accuracy.

## 5 Conclusion

In this retrospective cohort of patients with metastatic renal cell carcinoma treated with first-line ICI–TKI therapy, elevated baseline serum IgG was associated with significantly inferior survival outcomes, particularly OS, and remained an independent prognostic factor after adjustment for established clinical covariates. Baseline IgG still showed added prognostic value beyond the IMDC risk model and improved the discriminative performance of survival prediction, particularly for 3-year OS, although the nomogram should be considered exploratory pending external validation. Prospective multicenter studies are warranted to validate these results and to further clarify the biological significance of IgG in the immunotherapy era.

## Data Availability

The raw data supporting the conclusions of this article will be made available by the authors, without undue reservation.

## Supplementary material

Supplementary Table 1 Sensitivity analyses restricted to clear cell RCC and pembrolizumab-axitinib subgroups.

Supplementary Table 2 Three-group sensitivity analysis of baseline serum IgG.

Supplementary Table 3 Nested Cox regression analyses showing the incremental prognostic value of baseline serum IgG beyond inflammatory markers.

Supplementary Table 4 Discriminative performance of prognostic models for overall survival and incremental value of adding baseline serum IgG.

Supplementary Table 5 Bootstrap internal validation and performance of the prognostic model.

Supplementary Table 6 Assessment of proportional hazards assumption for IgG group using multiple methods.

Supplementary Figure 1 Restricted cubic spline curves showing the nonlinear relationship between baseline serum IgG and **(A)** overall survival and **(B)** progression-free survival.

Supplementary Figure 2 Kaplan–Meier survival curves for the three-group IgG stratification. **(A)** Overall survival. **(B)** Progression-free survival.

Supplementary Figure 3 Calibration plots of the prognostic model for predicting 1-, 2-, and 3-year overall survival. The dashed diagonal line represents ideal calibration. The solid black line indicates the apparent model performance, while the blue line represents the optimism-corrected calibration generated using 1000 bootstrap resamples.

Supplementary Figure 4 Schoenfeld residual plots for assessment of the proportional hazards assumption.
